# The Plant-Derived Glucocorticoid Receptor Agonist Endiandrin A Acts as Co-Stimulator of Colonic Epithelial Sodium Channels (ENaC) via SGK-1 and MAPKs

**DOI:** 10.1371/journal.pone.0049426

**Published:** 2012-11-13

**Authors:** Dana Kuntzsch, Theresa Bergann, Petra Dames, Anja Fromm, Michael Fromm, Rohan A. Davis, Matthias F. Melzig, Joerg D. Schulzke

**Affiliations:** 1 Department of Gastroenterology, Division of Nutritional Medicine, Charité, Campus Benjamin Franklin, Berlin, Germany; 2 Institute of Clinical Physiology, Charité, Campus Benjamin Franklin, Berlin, Germany; 3 Eskitis Institute, Griffith University, Brisbane, Queensland, Australia; 4 Institute of Pharmacy, Pharmaceutical Biology, Freie Universität Berlin, Berlin, Germany; Massachusetts General Hospital, United States of America

## Abstract

In a search for secondary plant compounds that bind to the glucocorticoid receptor (GR), the cyclobutane lignan endiandrin A was discovered from the rainforest tree *Endiandra anthropophagorum* Domin. Our present study aims to characterize the effect of endiandrin A on GR-dependent induction of colonic sodium transport. The effect of endiandrin A was analyzed in GR-expressing colonic HT-29/B6 cells (HT-29/B6-GR). GR transactivation and subcellular localization were investigated by reporter gene assay and immunofluorescence. Epithelial sodium channel (ENaC) was analyzed by qRT-PCR and by measuring amiloride-sensitive short-circuit current (I_sc_) in Ussing chambers. Endiandrin A (End A) has been identified as GR receptor binder. However, it did not cause significant GR transactivation as pGRE-luciferase activity was only 7% of that of the maximum effect of dexamethasone. Interestingly, endiandrin A had a significant impact on dexamethasone-dependent sodium absorption in cells co-exposed to tumor necrosis factor (TNF)-α. This was in part due to up-regulation of β- and γ-ENaC subunit expression. Endiandrin A potentiated GR-mediated transcription by increasing GR protein expression and phosphorylation. It inhibited c-Jun N-terminal kinase (JNK) activation induced by dexamethasone and/or TNF-α and increased levels of GR localized to the nucleus. Additionally, endiandrin A increased the serum- and glucocorticoid-induced kinase (sgk)-1 via activation of p38. Finally, the regulation of ENaC function by endiandrin A was confirmed in rat native colon. In conclusion, endiandrin A potentiates glucocorticoid-driven activation of colonic epithelial sodium channels via JNK inhibition and p38 activation due to transcriptional up-regulation of β- and γ-ENaC-subunits along with induction of sgk-1.

## Introduction

The amiloride-sensitive ENaC is located in the apical membrane of aldosterone-responsive tissues and plays a key role in electrogenic Na^+^ absorption in a variety of epithelia, including lung, distal nephron and distal colon [1], [2]. ENaC consists of three subunits, α, β, and γ. While in the nephron all three subunits are regulated via mineralocorticoid (MC) and/or glucocorticoid (GC) receptor stimulation, in the colon only the β- and γ-subunits are regulated and the α-subunit is constitutively expressed. Na^+^ transport is greatly enhanced when all subunits are assembled to form functional ENaC channels [2], [3].

Among patients with inflammatory bowel diseases (IBD), barrier disturbance and diarrhea are key symptoms, mainly attributed to high levels of pro-inflammatory cytokines. Down-regulation of intestinal sodium transporters, as the sodium-hydrogen exchanger NHE-1 and/or ENaC are possible explanations [4], [5]. In IBD-associated diarrhea, it was shown that β- and γ-ENaC subunit expression was suppressed by pro-inflammatory cytokines like TNF-α, resulting in an impaired electrogenic Na^+^ absorption [4], [6]. For treating IBD symptoms glucocorticoids are administered, because they suppress intestinal inflammation and also restore sodium absorption to normal. Furthermore, Sandle and co-workers observed that in patients with osmotic diarrhea treated with GCs an improvement in Na^+^ and water absorption could be induced very rapidly [7]. In an attempt to explain this, Bergann and co-workers characterized a synergistic interaction of GC and TNF-α in inducing ENaC-dependent Na^+^ absorption based on a p38 mitogen-activated protein kinase (MAPK)-dependent signaling mechanism [8].

Beyond anti-inflammatory and pro-absorptive effects, GCs show various and frequent – in part serious – side effects, especially in the long-term treatment (e.g. of IBD). Therefore, the search for novel remedies including plant derived steroids is highly desirable and currently in progress. In the field of alternative hormone replacement therapy, phytoestrogenes like the active ingredients of soy, black cohosh or chasteberry have even become popular as a low-risk medication against female hormonal disorders [9]. In contrast, only a few plant-derived GR modulators have been described so far as research within this field has just begun [10]–[12].

*Endiandra anthropophagorum* Domin is an evergreen plant belonging to the family Lauraceae and grows solely in the Australian rainforest of far north Queensland. The roots of *E. anthropophagorum* Domin contain several lignans, including the cyclobutane lignan endiandrin A (End A). Endiandrin A has been reported by Davis and co-workers to show potent activity in a GR binding assay [12]. Our present study aimed to characterize endiandrin A in more detail by assessing glucocorticosteroid effects on colonic ENaC function.

Our results show that while endiandrin A alone possesses no significant GR transactivation capacity, it can effectively increase Na^+^ absorption by activating colonic ENaC under inflammatory conditions, for example when TNF-α is present. We determined that endiandrin A stimulates sodium absorption by influencing MAPKs. This is the first report of an effect of a natural cyclobutane lignan on colonic ENaC regulation and of a steroid synergism.

## Materials and Methods

### Cell Culture and Reagents

The study was performed with confluent monolayers of the human colon carcinoma cell line HT-29/B6-GR, established in our laboratory and described earlier in detail [13]. Cells were cultured at 37°C in a humidified 5% CO_2_ atmosphere and seeded for experiments on Millicell PCF filters (3 µm; Millipore, Schwalbach, Germany) in RPMI1640 with 500 IU/ml G418 (Biochrom AG, Berlin, Germany), 10% fetal calf serum (FCS, Biochrom AG) and 1% penicillin-streptomycin (PAA Laboratories GmbH, Pasching, Austria). Experiments were performed after 7 days when cells have reached transepithelial resistance (TER) values of 300–500 Ω cm^2^. Twenty-four hours prior to the experiment, cells were cultivated in medium with 10% hormone-free FCS (Biochrom AG). The isolated substance endiandrin A of *Endiandra anthropophagorum* Domin was supplied by Dr. Rohan Davis (Eskitis Institute, Griffith University, Brisbane, Australia). Purity studies were performed using analytical C18 high-performance liquid chromatography (HPLC). Following analysis of the HPLC data and integration of all UV peaks at 210 nm End A was shown to have a purity of 98%. End A was dissolved in DMSO and used in a concentration of 20 µM, unless otherwise noted. Dexamethasone (Sigma-Aldrich, Steinheim, Germany) was used in a concentration of 1 µM in Ethanol. TNF-α (TEBU, Offenbach, Germany) was used in a concentration of 500 IU/ml or 10,000 IU/ml in PBS. Specific inhibitors of MAPK, the p38 MAPK inhibitor SB202190, the p42/44 extracellular signal-regulated kinase (ERK, ERK’s upstream kinase MEK1/2) inhibitor U0126 and the JNK MAPK inhibitor SP600125 (Sigma-Aldrich) were used in a concentration of 10 µM. For GR antagonization RU-486 (10 µM; Sigma-Aldrich) was used.

### Ethics Statement

This study was performed in strict accordance with the recommendations for the Care and Use of Laboratory Animals at the Campus Benjamin Franklin, Charité, Berlin. All experimental procedures were approved by the Landesamt für Gesundheit und Soziales-Berlin (Permit Number: T2205).

### Animals

Male albino Wistar rats (250–300 g) were killed by inhalation of CO_2_. The colon was removed and stripped of serosa, muscularis propria, submucosa and outer layer of muscularis mucosae (totally stripped preparation). For this study, the late distal colon was used.

### Electrophysiological Measurements

For short-circuit current measurements confluent HT-29/B6-GR monolayers or rat distal colon were mounted into Ussing chambers. The circulating bathing solution was composed of (mM) Na^+^140; Cl^–^123.8; K^+^5.4; Ca^2+^1.2; Mg^2+^1.2; HPO_4_^2–^2.4; H_2_PO_4_^–^0.6; HCO_3_^–^21; D(+)-glucose 10; glutamine 2.5; D (+)-mannose 10; β-OH-butyrate 0.5 and antibiotics piperacillin (50 mg/l) and imipenem (4 mg/l). The solution was gassed with 95% O_2_ and 5% CO_2_ at pH 7.4 and kept at 37°C. The area exposed to the 10 ml bathing solution on each side was 0.60 cm^2^ for cell filters and 0.54 cm^2^ for rat distal colon. Monitoring of I_SC_ (µA/cm^2^) and TER (Ω cm^2^) was carried out using programmable voltage clamp devices (CVC6 and CVC8, Fiebig, Berlin, Germany). For filter-grown cell experiments, cells were incubated with dexamethasone (1 µM), and/or TNF-α (500 IU/ml) and/or End A (20 µM) for 24 hours unless otherwise noted. After mounting into the Ussing chamber and an equilibration period for 20 minutes, amiloride (100 µM; Sigma, Germany) was added to the apical side of the cell monolayer. For measurements on native tissue, rat distal colon was used. Here, Ussing chamber studies could only performed for about 14 hours due to a limited tissue viability under these conditions which is assumed to result from the initiation of epithelial apoptoses in vitro. Therefore samples of rat distal colon were treated additionally with 1,000 IU/ml IFN-γ and a 20-fold higher TNF-α concentration than in cell culture experiments for inducing effects as early as possible in this set up. For measurements on rat distal colon, IFN-γ (1000 IU/ml; TEBU) was added to the serosal compartment of mounted colon for 1 hour, after an equilibration with heat-inactivated hormone-free FCS (10%) for 30 minutes to avoid adhesion of the cytokines on glass surface of the Ussing set-up. The pre-incubation step with IFN-γ served to enhance the sensitivity to TNF-α exposure in rat distal colon, as IFN-γ pre-treatment can increase TNFα receptor expression. Then, TNF-α (10,000 IU/ml) was added to the serosal compartment and, depending of the experiment, dexamethasone (1 µM) and/or End A (20 µM) were added to both sides for 14 hours. Experiments ended with addition of amiloride (10^−4^ M) to the mucosal compartment. After 10 minutes of amiloride addition the ENaC-dependent sodium ﬂux was determined as the drop in I_SC_.

### RNA Isolation, Reverse Transcription and Real-time Polymerase Chain Reaction

Total RNA extraction was performed using peqGOLD RNApure (PEQLAB, Biotechnologie GmbH, Germany) according to the manufacturer’s instructions and RNA was quantified by NanoDrop® ND-1000 UV-Vis Spectrophotometer (PEQLAB). 2 µg total RNA per reaction were used for cDNA synthesis using High Capacity cDNA Reverse Transcription Kit (Applied Biosystems, Mannheim, Germany) as described by the manufacturer. Real-time polymerase chain reaction (PCR) was performed on a 7500 FAST Real-time PCR System using TaqMan Gene Expression Assays No. HS00165722_m1, HS00168918_m1, HS00178612_m1 and HS00230818_m1 for human ENaC-β, -γ and human sgk-1 and human GR, respectively (Applied Biosystems). Glyceraldehyde-3-phosphate dehydrogenase (GAPDH, unlabeled primer, VIC/TAMRA dye-labeled probe; Applied Biosystems) served as an endogenous control used for normalization of the threshold cycle (Ct) values of the examined genes. Differential expression was calculated according to the 2^−ΔΔCT^ method.

### Transfection and Luciferase Assay

For luciferase assay, HT-29/B6-GR cells were seeded under hormone-free conditions to 6-well plates 24 hours before transfection with a density of 5×10^5^ cells/well. Transfection was carried out using Lipofectamine with Plus reagent (Invitrogen, Karlsruhe, Germany). pGRE-Luc (Clontech, Saint-Germain-en-Laye, France) containing glucocorticoid response element (GRE) sequences followed by a Fireﬂy luciferase (luc) gene served as a reporter gene vector for GRE-driven luciferase expression. A 500 bp promoter fragment −1577 to −1076 bp relative to the transcription start side of the serum- and glucocorticoid-induced kinase (sgk)-1 gene was amplified by PCR from human genomic DNA (Promega) and inserted upstream of the luciferase reporter gene in pGL4.23-Luc (Promega), using XhoI and HindIII restriction sides. This promoter fragment has been shown to contain functional MREs/GREs and is here referred to as pSGK-1-short-Luc [14], [15]. p GL4.70 [hRluc] (Promega, Mannheim, Germany) served as a co-reporter construct containing the Renilla luciferase gene and was used to normalize the transfection efficiency. For experiments 1 µg of the reporter constructs and 50 ng of the coreporter per well were added. Dexamethasone (1 µM), End A (20 µM) and/or TNF-α (500 IU/ml) unless otherwise noted were added to the cells 4 hours after transfection. For inhibitor experiments cells were incubated with inhibitor substances 1 hour earlier. Cells were incubated for 24 hours at 37°C and 5% CO_2_. Measurement of fireﬂy and Renilla luciferase activity was determined with the Dual-Luciferase Reporter Assay System (Promega) and Centro LB 960 microplate-luminometer (Berthold, Bad Wildbad, Germany) according to the manufacturer’s instructions.

### Western Blot Analysis

For analysis of the GR and p-GR, p38, ERK and JNK MAPK and p-MAPK and β-actin amount, whole cell protein was prepared from filter-grown HT-29/B6-GR cells by scraping cells into an ice-cold solution of 250 µl lysis buffer ((mM) TRIS 20, pH 7.5, NaCl 150, mM EDTA 1, PMSF 1, Triton X-100 1, sodium pyrophosphate 2.5, 1 mM β-glycerophosphate, sodium vanadate 1, EGTA 1, 1 µg/ml Leupeptin). To facilitate lysis of the cells, cells were pushed through a 1-ml hypodermic syringe with a 25-mm needle. Samples were left under constant agitation at 4°C for 5 minutes and whole cell extract was obtained as the supernatant of a final centrifugation (10 minutes at 18,000 rpm, 4°C). Protein concentration was determined by the BCA method, reagents have been purchased from Pierce (Perbio Science GmbH, Bonn, Germany). Protein samples (20 µg) were separated by SDS-polyacrylamide gel electrophoresis (8.5%) and blotted onto a PVDF-membrane according to standard protocols. After 2 hours blocking in 5% BSA/TBS-T, membranes were incubated overnight with primary antibodies: anti-β-actin (1∶10,000, Sigma), anti-phospho-p42/44 (anti-p-p42/44 MAPK (Thr202/Tyr204), 1∶1,000), anti-phospho-p38 (anti-p-p38 (Thr334), 1∶1,000); anti-p42/44 (anti-p42/44 MAPK (Thr202/Tyr204), 1∶1,000), anti-p38 (anti-p38 (Thr334), 1∶1,000); anti-phospho-SAPK/JNK (anti-p-SAPK/JNK (Thr183/Tyr185), 1∶1,000), anti-SAPK/JNK (anti-SAPK/JNK, 1∶1,000), (all antibodies: Cell Signaling). GR protein levels were analyzed with the primary antibodies anti-GR (anti-GR, H-300, 1∶200, Santa Cruz, Heidelberg, Germany) and anti-phospho-GR (anti-p-GR (Ser211), 1∶1,000). Here, only the functionally relevant GR-α protein was evaluated and in the following described as total GR protein levels. For densitometric analyzes of GR protein levels, samples were normalized to dexamethasone, as control samples could not be analyzed due to the low GR level. For protein detection LumiLight Plus Western blotting kit (Roche, Mannheim, Germany) containing POD-conjugated secondary antibodies and a chemiluminescence system was applied. Chemiluminescence signals were detected using an Fusion FX7 imaging system (Vilber Lourmat, Ebershardzell, Germany) and were analyzed with the AIDA program package (Raytest, Berlin, Germany).

### Immunofluorescence

Immunofluorescence staining with GR-antibody and DAPI by confocal laser scanning microscopy was performed as described earlier [8].

### Statistical Analysis

Data are expressed as means ± standard error of the mean (s.e.m.). For statistical analysis the 2-sided Student t-test was used. For comparison of more than 2 groups, Bonferroni-Holm correction was applied. *P*<0.05 was considered significant.

## Results

### GR Transactivation and Na^+^ Absorption under the Impact of Endiandrin A in Colonic HT-29/B6-GR Cells (Figure 1)

**Figure 1:**
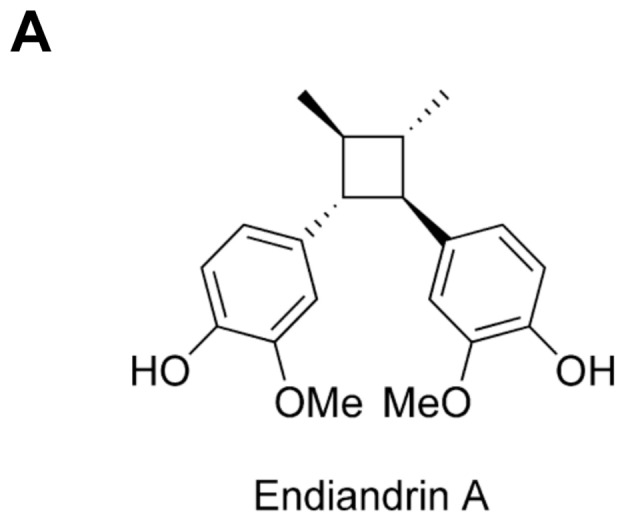
Effect of endiandrin A on GR transactivation in HT-29/B6-GR cells. (**A**) Structure of endiandrin A.

Cytotoxicity tests with the crystal violet staining method showed that endiandrin A has no cytotoxicity at the concentrations between 1 µM and 100 µM applied in this study (data not shown). As shown in Fig. 1B, endiandrin A induced no significant transactivation with respect to a GRE-driven luciferase reporter gene construct. As a reference, induction of luciferase activity by the synthetic glucocorticoid dexamethasone at 1 µM is given (2146±282%, Fig. S1A). Since luciferase activity appeared to decrease at higher doses, a concentration of 20 µM was used for the further experiments in this study.

In line with the results for GR transactivation, electrophysiological experiments revealed a tendency for endiandrin A to enhance ENaC-mediated Na^+^ absorption after 48 hours (0.29±0.06 µA/cm^2^), but again this did not reach statistical significance (Fig. S1B).

### Amplification of GC-induced ENaC-dependent Na^+^ Absorption under Inflammatory Conditions by Endiandrin A through Increased β- and γ-ENaC Expression in Colonic HT-29/B6-GR Cells (Figure 2)

**Figure 2:**
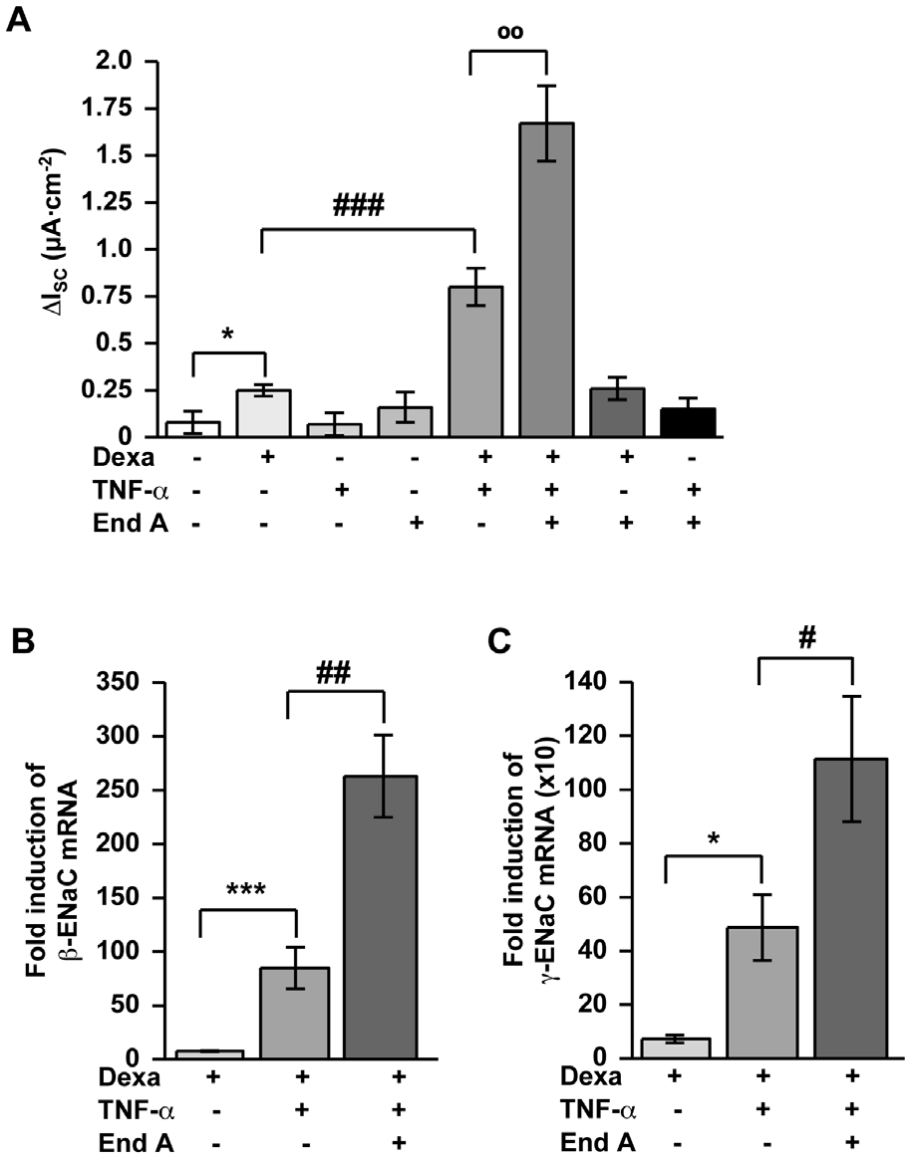
Enhancement of ENaC-dependent Na^+^ absorption and β- and γ-ENaC transcription by endiandrin A in HT-29/B6-GR cells. HT-29/B6-GR cells were incubated with dexamethasone (1 µM) and/or TNF-α (500 IU/ml) and/or endiandrin A (20 µM) for 24 hours. (**A**) Measurement of ENaC-dependent Na^+^ absorption was determined as the drop in I_SC_ after amiloride (100 µM) after 24 hours. Data are means ± s.e.m., n = 6–15, ^*^ *P*<0.05 compared with control, ^###^ *P*<0.001 compared with dexamethasone alone, ^°°^ *P*<0.01 compared with dexamethasone + TNF-α 500 IU/ml. (**B**)/(**C**) Measurement of (**B**) β- or (**C**) γ-ENaC-mRNA. GAPDH was used for normalization of mRNA expression. (**B**) Real-time PCR of β-ENaC mRNA after 24 hours. Data are means ± s.e.m., n = 4–13, ^***^ *P*<0.001 compared with dexamethasone alone, ^##^ *P*<0.01 compared with dexamethasone + TNF-α 500 IU/ml. (**C**) Real-time PCR of γ-ENaC mRNA after 24 h. Data are means ± s.e.m., n = 4–12, ^*^ *P*<0.05 compared with dexamethasone alone, ^#^ *P*<0.05 compared with dexamethasone + TNF-α 500 IU/ml.

To examine whether endiandrin A alters the interplay between GCs and TNF-α to increase ENaC-mediated Na^+^ absorption, HT-29/B6-GR cells were incubated with dexamethasone, TNF-α and endiandrin A for 24 hours and subsequently Ussing chamber experiments were performed. As expected, the treatment with dexamethasone led to a slight increase in ENaC-mediated Na^+^ absorption (0.25±0.03 µA/cm^2^, *P<0.05, compared with control), whereas Na^+^ absorption showed a strong increase after co-incubation with TNF-α and dexamethasone (0.80±0.10 µA/cm^2^, ^###^ *P*<0.001, compared with dexamethasone alone, Fig. 2A). Interestingly, additional exposure of dexamethasone- and TNF-α-treated cells to endiandrin A caused a marked enhancement of ENaC-mediated Na^+^ absorption (1.67±0.03 µA/cm^2^, °°*P*<0.01, compared with dexamethasone and TNF-α alone; Fig. 2A). To investigate whether this effect was accompanied by an increase in ENaC expression, we performed real-time PCR on β- and γ-ENaC. Our data reveal a stronger elevation of β- and γ-ENaC mRNA levels after co-incubation of HT-29/B6 cells with endiandrin A compared to dexamethasone and TNF-α treatment alone (263±38-fold of control for β-ENaC, ^##^ *P*<0.01, and 1114±233-fold of control for γ-ENaC, ^#^ *P*<0.05, versus dexamethasone and TNF-α alone, Figs. 2B and 2C). Concerning the α-ENaC subunit no change in expression level was found (data not shown). Nevertheless, the GR-antagonist RU-486 was used to verify that the observed effects reflect GR activity. After 1 hour pre-incubation with RU486, ENaC-mediated Na^+^ absorption as well as γ-ENaC subunit expression were significantly blocked (1.45±0.13 µA/cm^2^ and 894±191-fold for γ-ENaC mRNA for dexamethasone, TNF-α and endiandrin A; 0.54±0.11 µA/cm^2^ and 236±31-fold for γ-ENaC mRNA after RU486, dexamethasone, TNF-α and endiandrin A incubation, data are means ± s.e.m., n = 6–8, ^**^ *P*<0.01 and ^***^ *P*<0.001 compared to dexamethasone, TNF-α 500 IU/ml and endiandrin A without inhibitor, data not shown). Our findings suggest that endiandrin A potentiates the synergism between dexamethasone and TNF-α by enhancing ENaC-mediated Na^+^ absorption through an up-regulation of β- and γ-ENaC mRNA.

### Dexamethasone-induced GR Transactivation under Inflammatory Conditions Increases by Endiandrin A (Figure 3)

**Figure 3:**
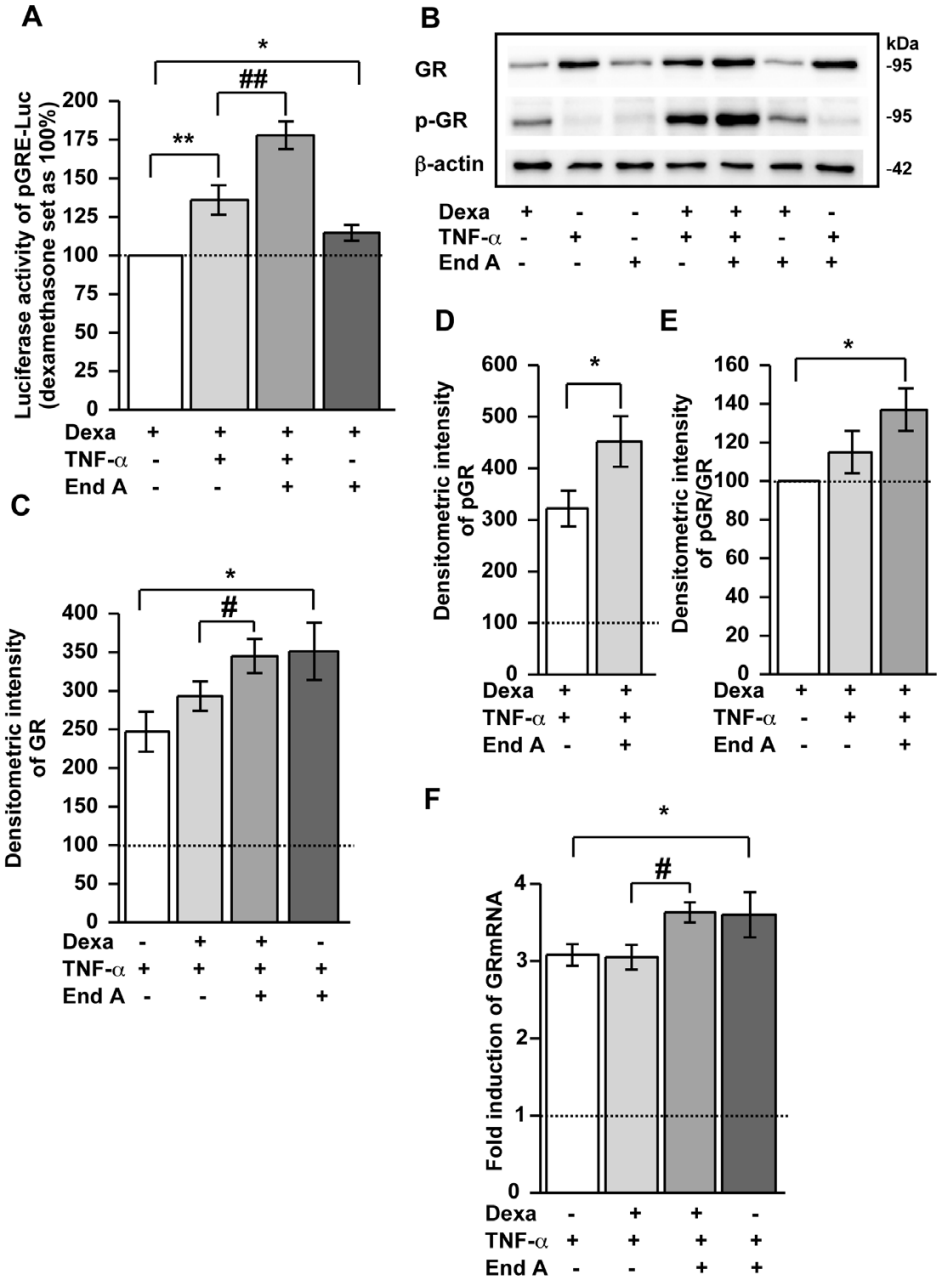
Endiandrin A increased activation of a GRE-driven luciferase construct and GR protein levels in HT-29/B6-GR cells. (**A**) HT-29/B6-GR cells were transfected with pGRE-Luc and incubated with dexamethasone (1 µM) and/or TNF-α (500 IU/ml) and/or endiandrin A (20 µM) for 24 hours. Data are given as normalized relative luciferase activity and as means ± s.e.m., n = 6–14 ^*^ *P*<0.05, ^**^ *P*<0.01 compared with dexamethasone alone, ^##^ *P*<0.01 compared with dexamethasone + TNF-α 500 IU/ml. (**B**) Western blot analysis of total GR and phospho-GR protein (∼95 kDa) of HT-29/B6-GR cell lysates incubated with dexamethasone (1 µM) and/or TNF-α (500 IU/ml) and/or endiandrin A (20 µM) for 3 hours. Human β-actin (∼42 kDa) served as a loading control. (**C**) Densitometry of total GR chemiluminescence signals. Shown are means ± s.e.m., n = 5, *P<0.05 compared to TNF-α alone, ^#^ *P*<0.05 compared with dexamethasone + TNF-α 500 IU/ml. (**D**) Densitometry of phospho-GR chemiluminescence signals. Shown are means ± s.e.m., n = 5, *P<0.05 compared with dexamethasone + TNF-α 500 IU/ml. (**E**) Densitometry of phospho-glucocorticoid receptor chemiluminescence signals normalized to total glucocorticoid receptor. Shown are means ± s.e.m., n = 5, *P<0.05 compared with dexamethasone. (**F**) GR mRNA expression of HT-29/B6-GR cells incubated with dexamethasone (1 µM) and/or TNF-α (500 U/ml) and/or endiandrin A (20 µM) for 3 hours. GR mRNA level was analyzed by real-time PCR. Data are means ± s.e.m. of x-fold induction of GR expression over that of untreated controls, n = 7–8, *P<0.05 compared to TNF-α alone, ^#^ *P*<0.05 compared with dexamethasone + TNF-α 500 IU/ml.

For investigating whether the increase in β- and γ-ENaC subunits under endiandrin A exposure depends on GR binding to GRE, HT-29/B6-GR cells were transiently transfected with a GRE-driven luciferase reporter gene construct and incubated with dexamethasone, TNF-α and endiandrin A. As expected, dexamethasone and TNF-α synergistically induced a strong increase in luciferase activity, which exceeds luciferase activity induced by dexamethasone alone (Fig. 3A). Surprisingly, reporter gene analyses revealed that endiandrin A amplified both dexamethasone-induced and dexamethasone- and TNF-α-induced luciferase activity, thus reflecting stronger GRE activation (Fig. 3A). Exposure to TNF-α or to endiandrin A alone showed no induction of luciferase activity.

Previous work by our group revealed that increased GR transactivation in response to TNF-α and dexamethasone could be traced back to an up-regulation of GR expression on mRNA as well as protein level due to TNF-α [8]. Consequently, in the present study we investigated whether enhanced GR activity was the result of an increase in GR expression levels. After 3 hours of incubation with dexamethasone, TNF-α and endiandrin A, GR protein expression was monitored by western blot analyses. As shown in Fig. 3B, GR protein levels increased as expected when cells were incubated with TNF-α (247±26%, ^*^ *P*<0.05 of dexamethasone level, Fig. 3C). Remarkably, GR protein levels increased additionally under endiandrin A treatment (351±37%, ^*^ *P*<0.05 compared to TNF-α alone, Fig. 3C). An increase of GR protein level was also observed when cells were incubated with endiandrin A in addition to dexamethasone and TNF-α (345±22%, ^*^ *P*<0.05 compared to TNF-α and dexamethasone, Fig. 3C), while treatment with TNF-α and dexamethasone led to no changes in GR levels compared to TNF-α. No changes in GR levels were also determined when cells were incubated with endiandrin A or dexamethasone combined with endiandrin A.

Wang and co-workers revealed that the extent of phosphorylated Ser211 of human GR is associated with GR transactivation [16]. The following set of investigations aimed at the determination of the Ser211-phosphorylated GR, since we found a stronger GRE activation and an enhanced GR protein level under endiandrin A treatment. As shown in Fig. 3B, dexamethasone induced Ser211-GR phosphorylation. Interestingly, co-incubation with endiandrin A, dexamethasone and TNF-α intensified Ser211-GR phosphorylation (452±49%, ^*^ *P*<0.05 versus dexamethasone and TNF-α, Fig. 3D) compared to TNF-α and dexamethasone alone (322±35%, Fig. 3D). Densitometric analysis revealed a different ratio of phosphorylated to total GR between exposure to dexamethasone alone or to dexamethasone with a combination of TNF-α and endiandrin A (137±11%, ^*^ *P*<0.05 compared to dexamethasone alone, Fig. 3E). The ratio of phosphorylated to total GR between the exposure to dexamethasone and TNF-α or to a combination of dexamethasone with a combination of TNF-α and endiandrin A showed a tendency towards an increase in activated GR. However, this did not reach statistical significance.

Furthermore, as shown in Fig. 3F we also determined GR mRNA expression after 3 hours of incubation with dexamethasone, TNF-α and endiandrin A by real-time PCR analyses. Under endiandrin A exposure a moderate elevation of GR mRNA was detectable compared to dexamethasone and TNF-α (3.5±0.1-fold of control for GR,^ #^ *P*<0.05 compared to dexamethasone and TNF-α alone, Fig. 3F). Endiandrin A was shown to up-regulate the TNF-α-induced increase in GR mRNA levels (3.6±0.3-fold,^ *^ *P*<0.05 compared to TNF-α alone, Fig. 3F), whereas endiandrin A or dexamethasone alone induced no change in GR mRNA expression.

Thus, endiandrin A potentiates GC-induced activated GR protein levels under inflammatory conditions through a synergism with TNF-α to increase GR mRNA and GR protein levels.

### Role of JNK Signaling for Enhanced ENaC-mediated Na^+^ Absorption (Figure 4)

**Figure 4:**
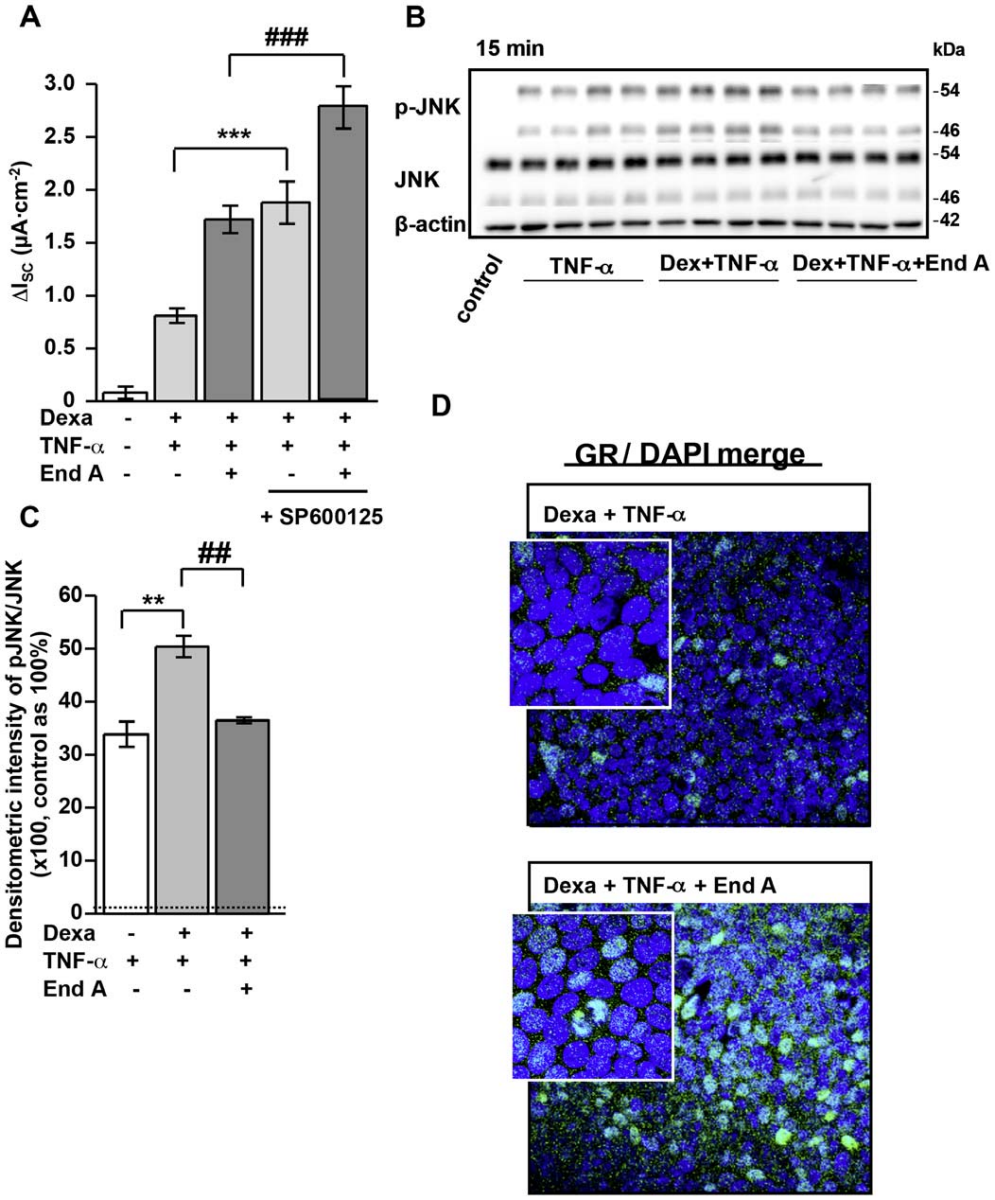
Enhanced nuclear GR translocation through inhibition of TNF-α-induced JNK phosphorylation by endiandrin A in HT-29/B6-GR cells. (**A**) HT-29/B6-GR cells were pre-incubated with the JNK inhibitor SP 600125 (10 µM) for 1 hour before addition of dexamethasone (1 µM) and/or TNF-α (500 IU/ml) and/or endiandrin A (20 µM) for 24 hours. Measurement of ENaC-dependent Na^+^ absorption occurred as the drop in I_SC_ after amiloride (100 µM). Data are given as means ± s.e.m., n = 10, ***P<0.001 compared with dexamethasone + TNF-α 500 IU/ml incubation, ^###^ *P*<0.001 compared to dexamethasone + TNF-α 500 IU/ml + endiandrin A. (**B**) Western blot analysis of JNK and pJNK protein (∼46/54 kDa) of HT-29/B6-GR cell lysates incubated with dexamethasone (1 µM) and/or TNF-α (500 IU/ml) w/wo endiandrin A (20 µM) for 15 minutes. Human β-actin (∼42 kDa) served as a loading control. (**C**) Densitometry of endiandrin A-induced effects on phosphorylated JNK levels normalized to total JNK. HT-29/B6-GR cells were incubated with dexamethasone (1 µM) and/or TNF-α (500 IU/ml) w/wo endiandrin A (20 µM) for 15 minutes. Shown are means ± s.e.m., n = 4, **P<0.01 compared to TNF-α exposure,^ ##^ *P*<0.01 compared to dexamethasone + TNF-α 500 IU/ml. (**D**) Immunofluorescence staining of GR. HT-29/B6-GR cells were incubated with dexamethasone (1 µM) and TNF-α (500 IU/ml) and/or endiandrin A (20 µM) for 3 hours. Cells were stained with anti-GR antibody (green), nuclei were DAPI stained (blue).

The JNK pathway is not directly implicated in the regulation of intestinal ENaC function, but several studies describe that JNK signaling can interfere with GR transactivation [17], [18]. Thus, we aimed to analyze the impact of endiandrin A on the JNK pathway. Therefore, the specific JNK blocker SP600125 was used and Ussing experiments were performed. Inhibition of JNK increased ENaC-mediated Na^+^ transport under dexamethasone and TNF-α as well as under their combination with endiandrin A (1.88±0.2 µA/cm^2^, ^***^ *P*<0.001 versus dexamethasone and TNF-α; 2.78±0.2 µA/cm^2^, ^***^ *P*<0.001 versus dexamethasone, TNF-α and endiandrin A, Fig. 4A). Furthermore we were interested whether endiandrin A influences JNK activation as monitored by means of JNK autophosphorylation. Endiandrin A alone had no significant effect on (unstimulated) JNK phosphorylation (Fig. S1C). On the other hand, endiandrin A was able to inhibit (stimulated) JNK phosphorylation, namely after 15 minutes TNF-α-dependent activation of the JNK (12113±1979% of control after TNF-α alone versus 6320±1063% of control after TNF-α and endiandrin A treatment, ^#^ *P*<0.05 versus TNF-α alone, Fig. S1C and D). Above inhibiting TNF-α-induced JNK phosphorylation, Western blot analyses showed endiandrin A also to inhibit TNF-α- plus dexamethasone-induced JNK phosphorylation after 15 minutes of stimulation (3384±239% of control after TNF-α alone versus 5041±200% of control after dexamethasone and TNF-α treatment, ^**^ *P*<0.01 compared to TNF-α alone; and 3648±56% of control after dexamethasone, TNF-α and endiandrin A treatment, ^##^ *P*<0.01 compared to dexamethasone and TNF-α treatment, Fig. 4B and 4C). Since JNK was reported to negatively influence GR transactivation and endiandrin A exposure inhibited JNK, we were interested whether or not endiandrin A induced nuclear translocation of the GR. Immunofluorescence staining of HT-29/B6-GR cells for the GR indicated that endiandrin A indeed increased GR nuclear staining (Fig. 4D).

### Role of p38 and ERK Signaling for Enhanced ENaC-mediated Na^+^ Absorption (Figure 5)

**Figure 5:**
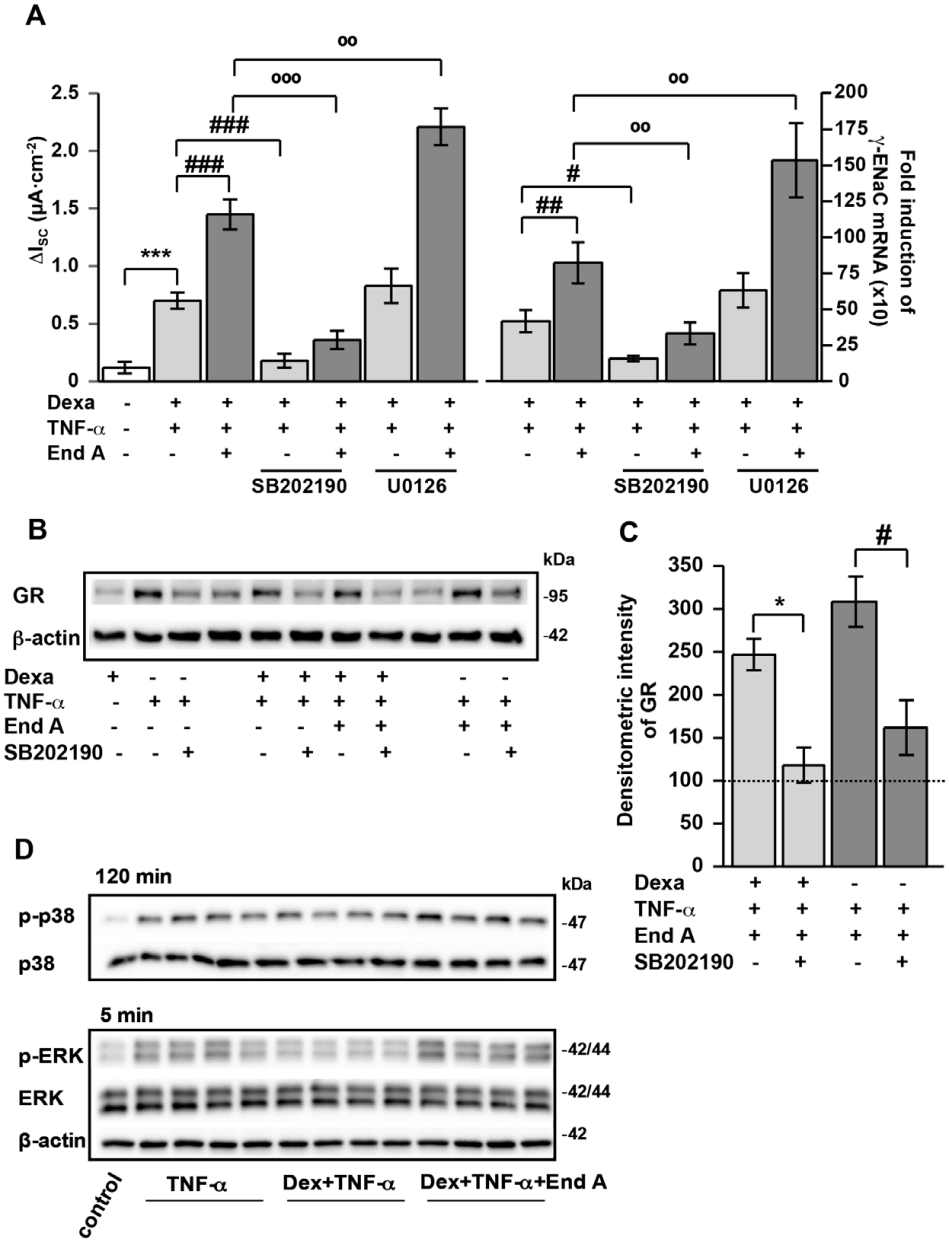
Synergistic ENaC induction by endiandrin A is linked to an activation of p38 and ERK in HT-29/B6-GR cells. HT-29/B6-GR cells were pre-incubated with the indicated MAPK inhibitors (10 µmol/L) for 1 hour before addition of dexamethasone (1 µM) and/or TNF-α (500 U/ml) and/or endiandrin A (20 µM) for 24 hours. (**A**) Measurement of ENaC-dependent Na^+^ absorption as the drop in I_SC_ after amiloride (100 µM) and measurement of γ-ENaC-mRNA after 24 hours. GAPDH was used for normalization of mRNA expression. Data are given as means ± s.e.m., n = 8–11 and n = 5–8, ***P<0.001 compared with dexamethasone incubation,^ #^ *P*<0.05, ^##^ *P*<0.01 and ^###^ *P*<0.001 compared to dexamethasone + TNF-α 500 IU/ml, ^°°^ *P*<0.01 and ^°°°^ *P*<0.001 compared with dexamethasone + TNF-α 500 IU/ml + endiandrin A. (**B**) Western blot analysis of total GR protein (∼95 kDa) of HT-29/B6-GR cell lysates. Cells were pre-incubated for 1 hour with SB202190 (10 µM) before incubation with dexamethasone (1 µM) and/or TNF-α (500 IU/ml) and/or endiandrin A (20 µM) for 3 hours. Human β-actin (∼42 kDa) served as a loading control. (**C**) Densitometry of SB202190 induced effects on total GR levels. Shown are means ± s.e.m., n = 7, *P<0.05 compared to dexamethasone, TNF-α 500 IU/ml and endiandrin A, ^#^ *P*<0.05 compared to TNF-α 500 IU/ml and endiandrin A. (**D**) Western blot analysis of HT-29/B6-GR cell lysates incubated with dexamethasone (1 µM) and/or TNF-α (500 IU/ml) w/wo endiandrin A (20 µM). Shown are p38 and pp38 (∼38 kDa) after 120 minutes stimulation as well as ERK and p-ERK MAPK protein (∼42/44 kDa) after 5 minutes stimulation. Human β-actin (∼42 kDa) served as a loading control.

Several studies indicated p38 and ERK to regulate Na^+^ transport [19], [20]. Therefore, p38 (SB202190) and ERK (U0126) inhibitors were used to investigate whether endiandrin A affected ENaC-dependent Na^+^ absorption via MAPK activation. Bergann and co-workers already showed that amiloride-sensitive short circuit current as well as γ-ENaC mRNA levels decreased when cells were treated with dexamethasone and TNF-α after pre-incubated with the p38 inhibitor SB202190 (Fig. 5A) [8]. Our results also indicated an impairment of ENaC-dependent Na^+^ absorption and γ-ENaC mRNA expression by pre-incubation with SB202190 indicating p38 to be involved in the endiandrin A-triggered ENaC-activation (0.58±0.10 µA/cm^2^, °°°*P*<0.001, and 437±100-fold for γ-ENaC mRNA, °°*P*<0.01 compared to dexamethasone, TNF-α and endiandrin A, Fig. 5A). Analysis of GR protein expression revealed that inhibition of p38 also blocks the TNF-α-dependent increase of GR protein levels (Fig. 4B). Furthermore, p38 inhibition also repressed the endiandrin A synergism to increase GR protein levels (53±5%, ^*^ *P*<0.05 compared to dexamethasone, TNF-α and endiandrin A and 62±6%, ^*^ *P*<0.05 compared to TNF-α and endiandrin A, Fig. 5B and 5C). Since TNF-α is an inducer of p38 activity, we tested the capability of endiandrin A to activate p38 MAPK and whether it acts synergistically with TNF-α. As shown in the supplemental data, TNF-α as well as endiandrin A led to a time-dependent phosphorylation of p38 MAPK. Densitometric analyses revealed that endiandrin A did not phosphorylate p38 at 5 minutes to the same extent as TNF-α, although p38 was phosphorylated to some extent after 15 minutes (440±40%, ^***^ *P*<0.001 versus control, Fig. S1E). Interestingly, this co-incubation of endiandrin A and TNF-α induced a prolonged p38 phosphorylation (335±56% of control after 120 minutes, ^#^ *P*<0.05 compared to TNF-α, Fig. S1E). Furthermore, we were interested whether endiandrin A could additionally prolong TNF-α- plus dexamethasone-induced p38 activation. Densitometric analyses revealed no significant alterations in p38 phosphorylation when cells were incubated for 120 minutes with dexamethasone plus TNF-α compared with TNF-α alone (Fig. 5D). However, additional exposure to endiandrin A prolonged p38 phosphorylation (526±40% of control after 120 minutes, n = 4, ^*^ *P*<0.05 compared to dexamethasone and TNF-α, Fig. 5D).

Then, we tested whether endiandrin A activates ERK signaling and whether inhibition of ERK can influence the increase of ENaC-dependent Na^+^ absorption. As already shown by Bergann and co-workers [8], amiloride-sensitive short circuit current as well as γ-ENaC mRNA levels showed no significant alterations when cells were treated with dexamethasone and TNF-α after pre-incubation with the ERK inhibitor U0126 (Fig. 5A). On the other hand, additional exposure to endiandrin A surprisingly led to an increase in ENaC-dependent Na^+^ transport, β-ENaC (data not shown) and γ-ENaC mRNA (2.96±0.35 µA/cm^2^, °°*P*<0.01, 1655±104-fold for γ-ENaC mRNA, °°*P*<0.01 compared to dexamethasone, TNF-α and endiandrin A, Fig. 5A). Densitometry of western blots revealed a rapid and strong phosphorylation but no prolonged ERK phosphorylation by endiandrin A alone (409±16% of control after 5 minutes, ^*^ *P*<0.05 compared to TNF-α alone, Fig. S1E). Furthermore, when cells were incubated for 5 minutes with dexamethasone and TNF-α, Western blots showed an inhibiting effect of dexamethasone on TNF-α-induced ERK activation (188±16% of control after TNF-α alone versus 125±5% of control after dexamethasone and TNF-α treatment, n = 4, ^*^ *P*<0.05 compared to TNF-α alone, Fig. 5D). On the contrary, additional endiandrin A stimulation intensified ERK phosphorylation (249±15% of control after dexamethasone, TNF-α and endiandrin A treatment, n = 4, ^##^ *P*<0.01 compared to dexamethasone and TNF-α, Fig. 5D).

Taken together, these results indicate a synergistic and prolonged p38 phosphorylation and furthermore an expeditious ERK activation under endiandrin A exposure.

### Sgk-1 Enhanced ENaC-dependent Na^+^ Absorption (Figure 6)

**Figure 6:**
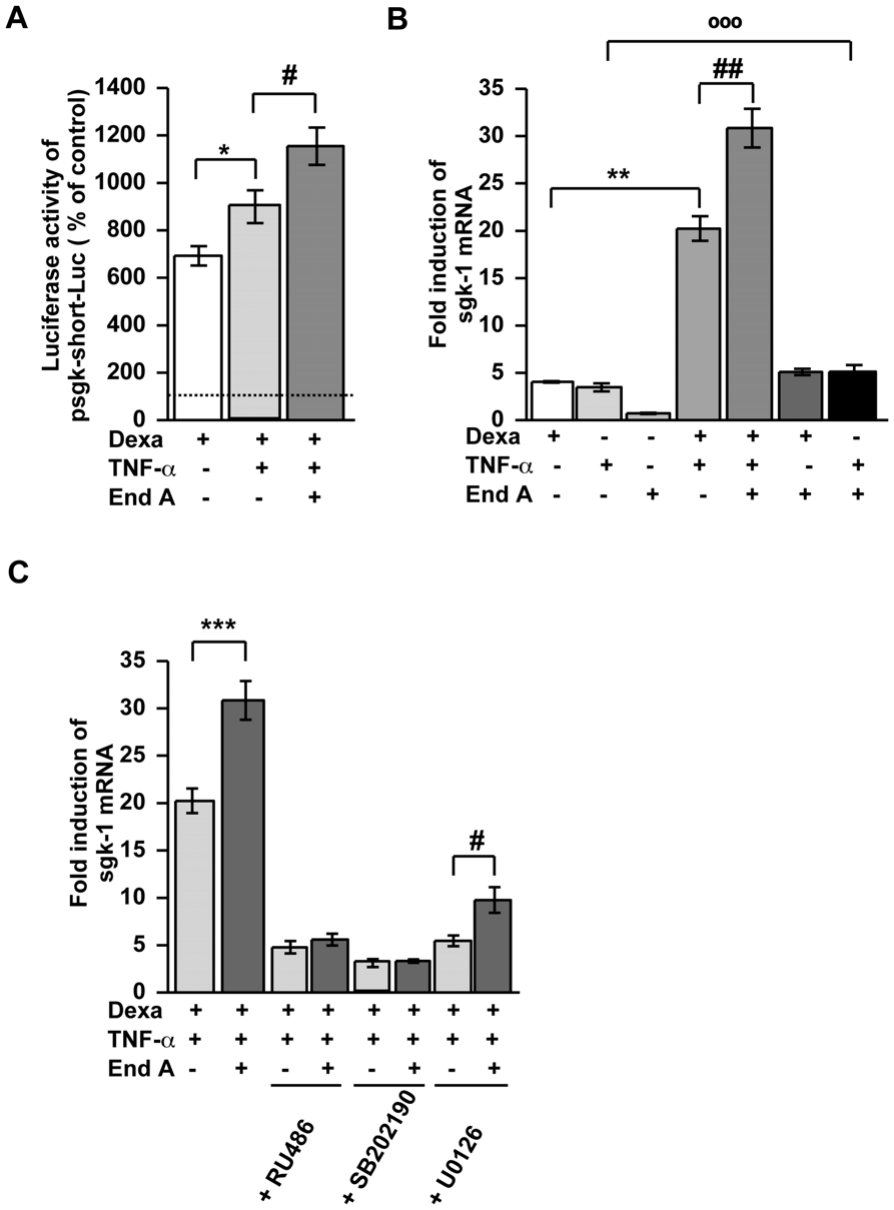
Influence of Sgk-1 on the synergistic ENaC induction in HT-29/B6-GR cells. (**A**) HT-29/B6-GR cells were transfected with psgk-1-short-Luc and incubated with dexamethasone (1 µM) and/or TNF-α (500 IU/ml) and/or endiandrin A (20 µM) for 24 hours. Data are given as normalized relative luciferase activity and as means ± s.e.m., n = 6–14 ^*^ *P*<0.05 compared to dexamethasone alone,^ #^ *P*<0.05 compared with dexamethasone + TNF-α 500 IU/ml. (**B**) Measurement of sgk-1 mRNA after 24 h. GAPDH was used for normalization of mRNA expression. Data are means ± s.e.m., n = 5–8, ^**^ *P*<0.01 compared with dexamethasone treatment, ^##^ *P*<0.01 compared with dexamethasone + TNF-α 500 IU/ml, ^°°°^ *P*<0.001 compared with TNF-α 500 IU/ml alone. (**C**) Measurement of sgk-1 mRNA pre-incubation with the indicated inhibitors (10 µM) for 1 hour before addition of dexamethasone (1 µM) and/or TNF-α (500 IU/ml) and/or endiandrin A (20 µM) for 24 hours. Data are means ± s.e.m., n = 5–7, ^***^ *P*<0.001 compared with dexamethasone + TNF-α 500 IU/ml, ^#^ *P*<0.05 compared to dexamethasone + TNF-α 500 IU/ml + U0126 inhibitor.

Serum- and GC-regulated kinase 1 (sgk-1) is a crucial mediator of MC- and GC-induced Na^+^ absorption via ENaC [14], [21]. Thus, we investigated whether sgk-1 is involved in the synergism of dexamethasone, TNF-α and endiandrin A. Therefore, a reporter gene construct with the sgk-1 promoter was transiently transfected into HT-29/B6-GR cells and it’s responsiveness to dexamethasone, TNF-α and endiandrin A treatment was monitored. As expected, dexamethasone induced a transcriptional activation of the sgk-1-reporter (Fig. 6A). Simultaneous incubation with TNF-α further increased promoter activity, which was even further amplified by additional endiandrin A exposure. Treatment with TNF-α or endiandrin A alone induced no promoter activation (data not shown). Additionally, to confirm the functionality of this enhanced activation of the sgk-1 promoter, sgk-1 mRNA expression levels were investigated by real-time PCR. For this purpose, HT-29/B6-GR cells were incubated with dexamethasone, TNF-α and endiandrin A for 24 hours. We found an increase of sgk-1 mRNA after dexamethasone as well as after TNF-α treatment, which was amplified when both were combined (20±1.3-fold of control, ^**^ *P*<0.01 versus dexamethasone alone, Fig. 6B). Interestingly, sgk-1 mRNA levels were further increased by additional endiandrin A treatment (31±2-fold of control, ^##^ *P*<0.01 versus dexamethasone and TNF-α treatment, Fig. 6B). Dexamethasone and endiandrin A showed a tendency towards an increase in sgk-1 mRNA levels when compared to dexamethasone exposure alone. However, this did not reach statistical significance. Surprisingly, co-incubation of TNF-α and endiandrin A increased sgk-1 mRNA expression in comparison to TNF-α alone (3.9±0.4-fold of control for TNF-α incubation and 6.0±0.5-fold of control for TNF-α combined with endiandrin A, °°°*P*<0.001, compared to TNF-α treatment, Fig. 6B). Specific blockers for GR (RU486), p38 (SB202190) and ERK MAPK (U0126) were used to elucidate signaling pathways behind this synergism of dexamethasone, TNF-α and endiandrin A to increase sgk-1 expression. As shown in Fig. 6C, sgk-1 mRNA was inhibited by the GR inhibitor RU486 and by the p38 inhibitor SB202190. In contrast, the ERK inhibitor U0126 inhibited sgk-1 expression, but the inhibition was significantly lower during endiandrin A co-exposure (9.8±1.3-fold of control, ^*^ *P*<0.05, versus dexamethasone, TNF-α and U0126 treatment, Fig. 6C). Our results indicate that the endiandrin A impact on sgk-1 occurs via the GR and via p38, respectively.

### Endiandrin A Amplifies GC- and TNF-α-induced Na^+^ Absorption via the ENaC also in Rat Distal Colon (Figure 7)

**Figure 7:**
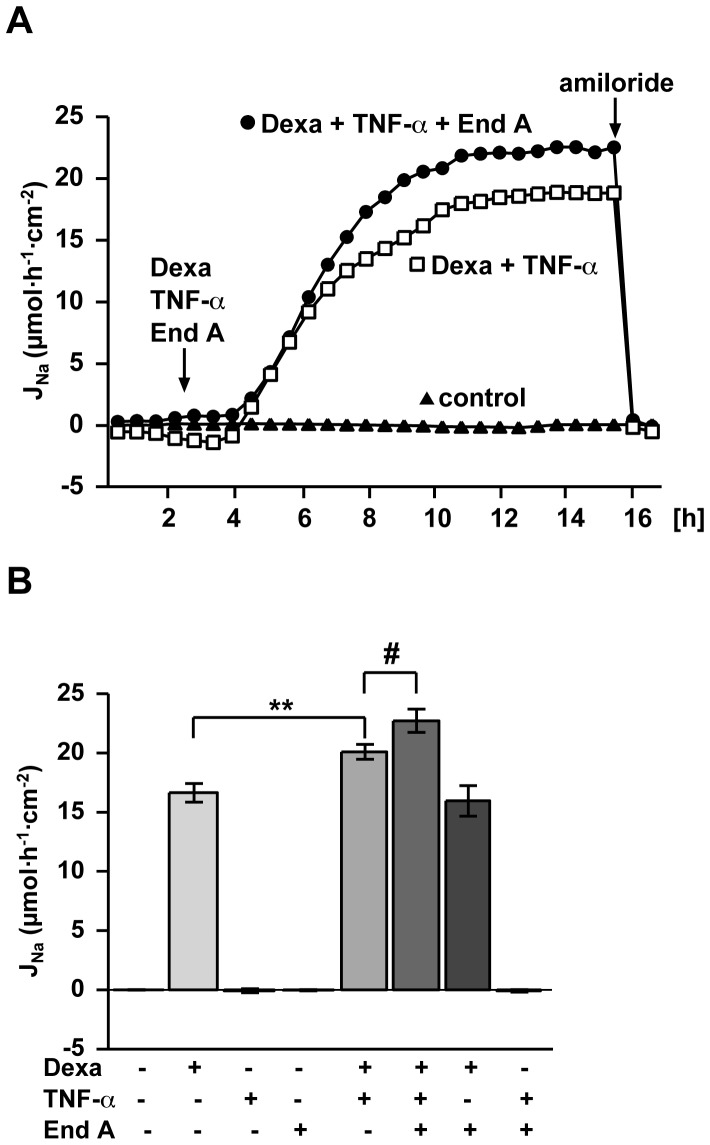
Endiandrin A amplified dexamethasone-induced ENaC-dependent Na^+^ absorption under inflammatory conditions in rat distal colon. (**A**) Time course of electrogenic Na^+^ transport in rat distal colon. Experiments were performed as described under methods. (**B**) Electrogenic Na^+^ transport in rat distal colon. Tissue was mounted into Ussing chambers and stimulated with dexamethasone (1 µM) and/or TNF-α (10,000 IU/ml) and/or endiandrin A (20 µM) for 14 hours after a pre-incubation with IFN-γ (1,000 IU/ml) for 1 hour. ENaC-dependent Na^+^ absorption was determined as the drop in I_SC_ within 10 minutes after addition of amiloride (100 µM). Results are given as means ± s.e.m., n = 4–18, **P<0.01 compared with dexamethasone treatment, ^#^ *P*<0.05 compared with dexamethasone + TNF-α 10,000 IU/ml.

To study whether endiandrin A amplifies the dexamethasone plus TNF-α up-regulated ENaC function also in native intestinal tissue, the effect of a triple-combination of endiandrin A, dexamethasone and TNF-α was investigated in rat distal colon in Ussing chambers as described previously [8] (Fig. 7A and B). In accordance with our results on HT-29/B6-GR cells, incubation with TNF-α and endiandrin A did not induce any change in amiloride-sensitive short circuit current, whereas dexamethasone treatment substantially increased electrogenic Na^+^ transport (16.6±0.8 µmol h^−1^ cm^−2^, Fig. 7B). As already observed by Bergann and co-workers before, co-incubation with TNF-α led to an induction of ENaC-mediated Na^+^ absorption (20.1±0.6 µmol h^−1^ cm^−2^, ^**^ *P*<0.01 versus dexamethasone alone, Fig. 7B) [8]. Na^+^ transport was further intensified by endiandrin A (22.7±1.0 µmol h^−1^ cm^−2^, ^#^ *P*<0.05 compared to dexamethasone plus TNF-α, Fig. 7B). This result indicates that endiandrin A affects Na^+^ transport not only in HT-29/B6-GR cells but also in rat native colon.

## Discussion

**Figure 8:**
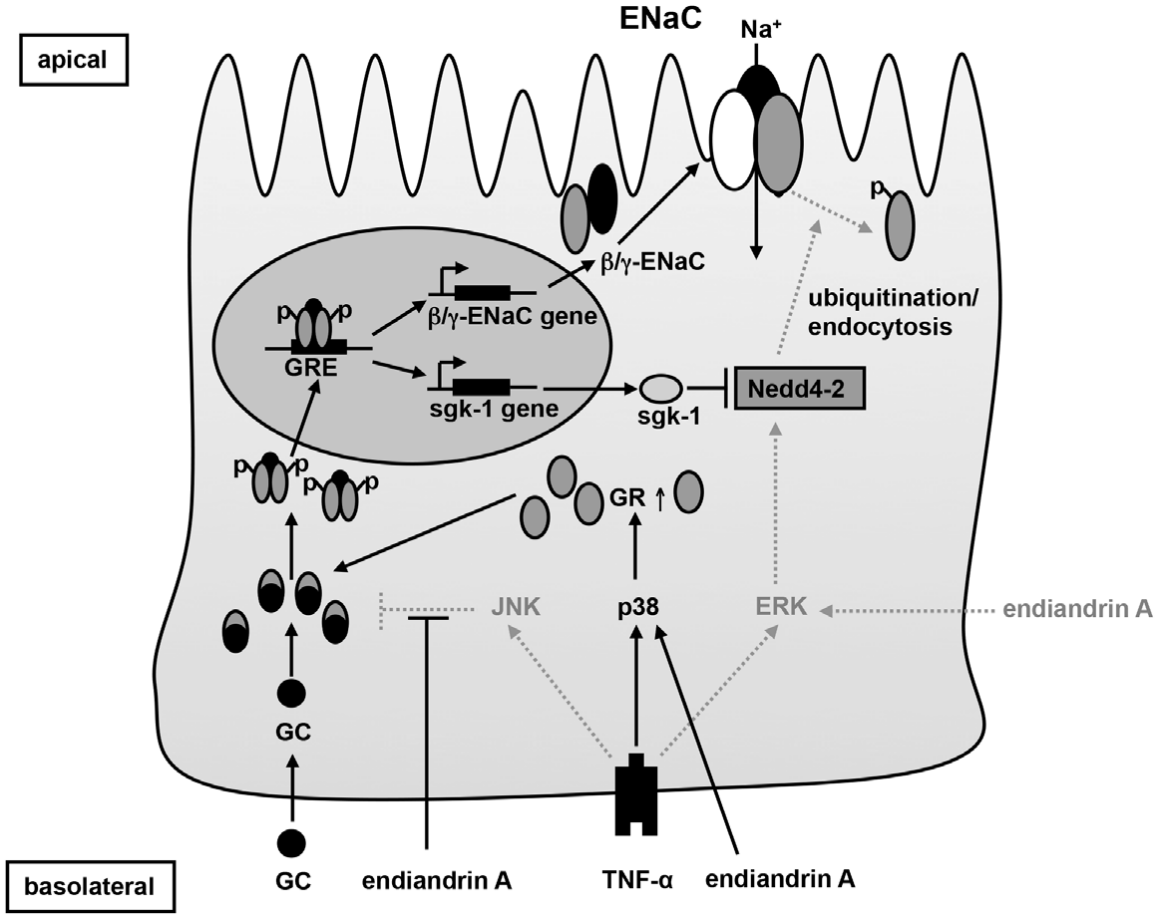
Scheme of endiandrin A action on ENaC-dependent Na^+^ absorption. p38, ERK and JNK are involved in the synergism of endiandrin A to impact colonic ENaC function. TNF-α-triggered JNK activation, which is described to negatively regulate the GR, is suppressed by endiandrin A. Furthermore, together with TNF-α, endiandrin A induces a prolonged p38 phosphorylation, which stabilizes GR mRNA and increases GR protein levels. Endiandrin A facilitates the dexamethasone-induced GR nuclear translocation resulting in an enhanced GRE-transactivation and an increase of β- and γ-ENaC as well as sgk-1 expression. Endiandrin A activates ERK, which impinges on the ENaC. Dotted lines indicate signaling pathways inhibiting the colonic ENaC function.

Endiandrin A was discovered due to its glucocorticoid receptor (GR) binding affinity [12]. However, so far it has not been determined whether endiandrin A also activates the GR in vitro or in vivo. Thus, our study was performed to investigate the GR functionality of endiandrin A. We demonstrated that endiandrin A does not only bind to the GR but also impacts ENaC function, in the human colorectal cancer cell line HT-29/B6-GR as well as in rat distal colon. These endiandrin A effects on ENaC regulation as well as the signaling pathways involved in this regulation are illustrated in Fig. 8.

### The Glucocorticoid Receptor and Endiandrin A

Activation of the GR is followed by receptor translocation and specific interferences with DNA sequences (glucocorticoid response elements, GREs). This has been shown to lead to transactivation and/or transrepression of target genes [22], [23]. Current research is focusing on the characterization of novel GC’s still exhibiting anti-inflammatory effects but with reduced side-effects, mainly triggered by the transactivation of the GR [24]. Possible candidates include plant-derived GR binder, only a few of which have been described so far. Ginsenoside-Rg1 (G-Rg1), a saponin compound from *Panax ginseng* C.A.MEY, was shown to bind and activate the GR in vitro as well as in vivo [10], [25]. Also Saikosaponin D (SSd), a saponin of *Bupleurum falcatum* L., was characterized as a GR binder in human epithelial carcinoma cells (HeLa) S3 cells [11]. Haridas and co-workers identified the plant triterpenoid, avicin D, as selective GR modulator [26]. All of these 3 plant compounds possess a similar GC-specific structure, which may explain theirs GR-agonistic effects, whereas endiandrin A seems to be a special GR binder with an unusual structure. But when modeling the endiandrin A-GR interaction, endiandrin A turned out to be a potential binder to the GR due to its stereochemistry (data not shown).

Wang and co-workers reported that the extent of phosphorylation of Ser211 in the human GR is associated with GR transactivation [16]. Although endiandrin A does induce GR-driven transcription only to a small degree, our results otherwise indicate that endiandrin A triggers phosphorylation of Ser211-GR. This is controversial on the first view, but Wang and co-workers also hypothesized that Ser211 phosphorylation alters GR confirmation finally facilitating transactivation. We suggest that endiandrin A co-activates the GR and thus simultaneously intensifies GCs effects, as we observed an increase in activated Ser211-GR protein levels after GC-induction. In addition, results of the gene reporter assay revealed that exposure to endiandrin A enhances the GC-induced activation of a glucocorticoid response element (GRE) indicating an intensified transactivation. Thereby we found that endiandrin A is able to increase ENaC-mediated Na^+^ absorption through an up-regulation of β- and γ-ENaC mRNA.

### Role of JNK in Endiandrin A-dependent Signaling

The c-Jun N-terminal kinase (JNK) is a member of the MAPK family that is activated by cytokines and cellular stress. Several studies describe that the activated JNK interferes with GR transcriptional activity through GR phosphorylation as well as posttranslational modifications [17], [18]. JNK was reported to phosphorylate Ser-246 of the human GR, thereby inhibiting its function and translocation [17]. In chronic inflammatory diseases a GC resistance is often found and many patients not respond to a GC therapy. Here the activated inflammatory pathways, especially the JNK pathway, may involve in a development of the resistance. Wang and co-workers described JNK inhibition as an therapeutic target for normalization of GR function [18]. As a result of our study, ENaC-mediated Na^+^ transport under GC and TNF-α treatment was enhanced by JNK inhibitor SP600125 and resembled that under endiandrin A during GC and TNF-α treatment. Immunofluorescence staining for the GR, also showed that under endiandrin A treatment, an increase of nuclear GR staining was detectable. Thus our data suggests that endiandrin A – similar to SP600125– inhibits JNK activation leading to an increase in GR translocation which facilitates the synergism of GC and TNF-α. In addition to endiandrin A, another plant-derived JNK inhibitor is described. Curcumin, a polyphenolic compound from *Curcuma longa* L., was characterized to inhibit JNK phosphorylation as well. Mun and co-workers showed that curcumin suppressed TNF-α-induced JNK activation in human fibroblast-like synoviocytes (FLSs) and SW1353 (a human chondrosarcoma cell line) cells leading to an amelioration of collagen-induced arthritis [27]. Interestingly, endiandrin A and curcumin posses an almost similar chemical structure as both of them have the same aromatic substituents. Further investigations are needed to clarify to what extent the chemical structure is crucial for the JNK inhibition.

### Role of p38 and ERK

For GCs therapies, a pro-absorptive effect was observed as the result of a synergism between GCs and TNF-α [7], [8]. Here, TNF-α was shown to increase GR protein levels by stabilizing GR mRNA via the p38 MAPK pathway. Noteworthy, stabilization of different mRNA targets was often observed after an activation of the p38 pathway [28], [29]. In human FLSs, the cytokine-induced stabilization of interleukin (IL)-6 mRNA was inhibited by SB202190, a specific p38 MAPK inhibitor [30]. Importantly, endiandrin A activates the p38 pathway in a time-dependent manner, which was synergistically amplified by dexamethasone and TNF-α. As a consequence, GR content was increased both on mRNA and protein level, which facilitates GCs effects. Although not tested here, endiandrin A could inhibit the MAPK phosphatase (MKP)-1 leading to prolonged p38 phosphorylation as shown by others [31], [32]. Such a mechanism is also described by Vogt and co-workers who identified the plant compound sanguinarine, an alkaloid from *Chelidonium majus* L., as an inhibitor of MPK-1 in HeLa cells [33].

In addition to p38, also the MAP kinase ERK can influence ENaC function [19], [34]. It was shown that ERK-dependent phosphorylation of the β- and γ-ENaC subunit decreases electrogenic Na^+^ absorption by facilitating the interaction with the ubiquitin ligase Nedd4-2 which targets ENaC complexes for endocytosis/degradation [19], [35]. We propose that the rapid activation of ERK after endiandrin A treatment, which is the next essential finding of our study, hampers electrogenic Na^+^ absorption via the ligase Nedd4-2. This mechanism may hide stimulating effects of endiandrin A on sodium absorption, e.g. via p38, because inhibition of ERK (by the selective MEK1/2 inhibitor U0126) increased ENaC-mediated Na^+^ transport as well as β- and γ-ENaC expression. Already Cobb and co-workers have characterized such a general interplay between ERK and p38 [36].

### Role of Sgk-1

We found a remarkable increase of the serum- and GC-regulated kinase (sgk)-1 pointing to a contribution of the ubiquitin ligase Nedd4-2 due to endiandrin A. As is generally accepted, the serine/threoine kinase sgk-1 inactivates Nedd4-2 through phosphorylation, subsequently preventing ubiquitination/endocytosis via Nedd4-2 [21], [37]. Previous studies observed an increase in sgk-1 mRNA as the consequence of corticosteroid action, which stimulated ENaC-mediated Na^+^ absorption in renal collecting duct cells [21], [38]. Interestingly, Itani and co-workers found that GCs stimulate sgk-1 expression via activation of a GRE in the 5′-flanking region of the sgk-1 gene [14]. Our data support this as we observed a strong increase in sgk-1 mRNA after GC treatment which was enhanced by TNF-α and which was even further intensified by endiandrin A.

Moreover, sgk-1 was reported to be influenced by p38. Waldegger and co-workers showed that p38 activation increased sgk-1 expression in the human hepatoma cell line HepG2 [39]. Furthermore, sgk-1 promoter activity was shown to be enhanced through p38 [40]. In our study, p38 inhibition (SB202190) decreased sgk-1 expression, which may have contributed to the decrease in ENaC-mediated Na^+^ absorption due to ubiquitination of ENaC-subunits via Nedd4-2. On the other hand, this cannot explain the decrease in γ-ENaC mRNA, which we found after SB202190 treatment. SB202190 also down-regulated GR protein levels. Therefore, we conclude that the inhibition of ENaC-mediated Na^+^ transport results from both of these 2 signaling events. Additionally, we found an increase of sgk-1 mRNA after TNF-α treatment, which could be amplified by endiandrin A. This may indicate sgk-1 mRNA stabilization and is in line with other reports e.g. of Meng and co-workers who reported an activation of sgk-1 which was triggered via the p38 pathway in biliary tract [41].

However, not only p38 influences sgk-1. Also ERK can activate sgk-1 [42]. On the other hand, over-expression of sgk-1 was recently reported to enhance ERK phosphorylation in rat primary hepatocytes [43]. Our present data indicates a decline in sgk-1 expression when incubated with the ERK inhibitor U0126, indeed pointing to a functional cross-talk between sgk-1 and ERK. Interestingly, the U0126-induced decrease in sgk-1 in our study did not cause any inhibitory effect on ENaC-mediated Na^+^ absorption or on γ-ENaC mRNA levels, which on the contrary rather increased because of the supposed enhanced p38 activation mentioned above. Thus, sgk-1 is influenced by the GR, p38 and ERK and is important for the synergism with endiandrin A. Kim and co-workers described sgk-1 as a negative regulator of JNK in the human embryonic kidney cell line 293T [44]. This is in line with the enhanced sgk-1 expression under GC and TNF-α, which may have led to inhibition of JNK activation also in our study. Additionally, this would be amplified by endiandrin A co-exposure.

### Steroid Therapy in Inflammatory Bowel Diseases (IBD) and Potential Role of Endiandrin A

Long-term GC treatment e.g. in IBD is always accompanied by undesirable side effects, including osteoporosis, Cushing’s syndrome and diabetes [45]. Here, phytopharmacologic approaches may be attractive. Phytopharmacological effects are not necessarily linked to GR activation, but may interfere with other signaling pathways, e.g. the NFκB-pathway. Catapoloside, a major iridoid glycoside isolated from *Catalpa ovata* G.DON, widely used in Korean medicine for colonic inflammation, has been shown to down-regulate NFκB-signaling [46]. Others have described anti-inflammatory effects of curcumin, a diferulolymethane from *Curcuma longa* L., or oleogum resins of *Boswellia* species [47], [48]. Only a few plant-derived GR modulators are characterized so far such as G-Rg1 and SSd. Interestingly, neither G-Rg1 nor SSd influenced colonic ENaC function in our experimental system (data not shown).

Certainly, endiandrin A cannot replace GC therapy in IBD. But endiandrin A may either amplify physiological cortisol effects or may at least help to reduce the dose of a therapeutic steroids in a general attempt to reduce steroid side effects by multimodal therapies.

In conclusion, we have determined the GC-agonistic effects of a main component of *E. anthropophagorum* Domin, endiandrin A, in HT-29/B6-GR cells as well as in rat distal colon and found a novel regulatory influence of this plant-derived GR binder via p38, ERK and JNK and sgk-1, resulting in synergistic effects with GC and TNF-α to impact colonic ENaC function.

## Supporting Information

Figure S1 (**Fig.** **S1A**) HT-29/B6-GR cells were transfected with pGRE-Luc and incubated with endiandrin A (1, 10, 20 and 100 µM) for 24 hours. Incubation with dexamethasone (1 µM) alone was performed as positive control. Data are given as normalized relative luciferase activity and as means ± s.e.m., n = 4–6. (**Fig.** **S1B**) Measurement of ENaC-dependent Na^+^ absorption was determined as the drop in I_SC_ after amiloride (100 µM). HT-29/B6-GR cells were incubated with endiandrin A (20 µM) for 48 hours. Data are means ± s.e.m., n = 6–12. (**Fig.** **S1C**) Western blot analysis of JNK and pJNK protein (∼46/54 kDa) of HT-29/B6-GR cell lysates incubated with TNF-α (500 IU/ml) and/or endiandrin A (20 µM) without dexamethasone for indicated times. Human β-actin (∼42 kDa) served as a loading control. (**Fig.** **S1D**) Densitometry of endiandrin A-induced effects on phosphorylated JNK levels normalized to total JNK. HT-29/B6-GR cells were incubated with TNF-α (500 IU/ml) and/or endiandrin A (20 µM) for 15 minutes. Shown are means ± s.e.m., n = 5, *P<0.05 compared to TNF-α exposure. (**Fig.** **S1E**) Western blot analysis of p38 and pp38 (∼38 kDa) as well as ERK and p-ERK MAPK protein (∼42/44 kDa) of HT-29/B6-GR cell lysates incubated with TNF-α (500 IU/ml) and/or endiandrin A (20 µM) without dexamethasone for indicated time points. Human β-actin (∼42 kDa) served as a loading control.
(TIF)
