# Supplementary figures and images for: The Plant-Derived Glucocorticoid Receptor Agonist Endiandrin A Acts as Co-Stimulator of Colonic Epithelial Sodium Channels (ENaC) via SGK-1 and MAPKs
Source: PLoS One. 2012 Nov 13;7(11):e49426. doi: 10.1371/journal.pone.0049426 (PMC3496671; doi:10.1371/journal.pone.0049426)

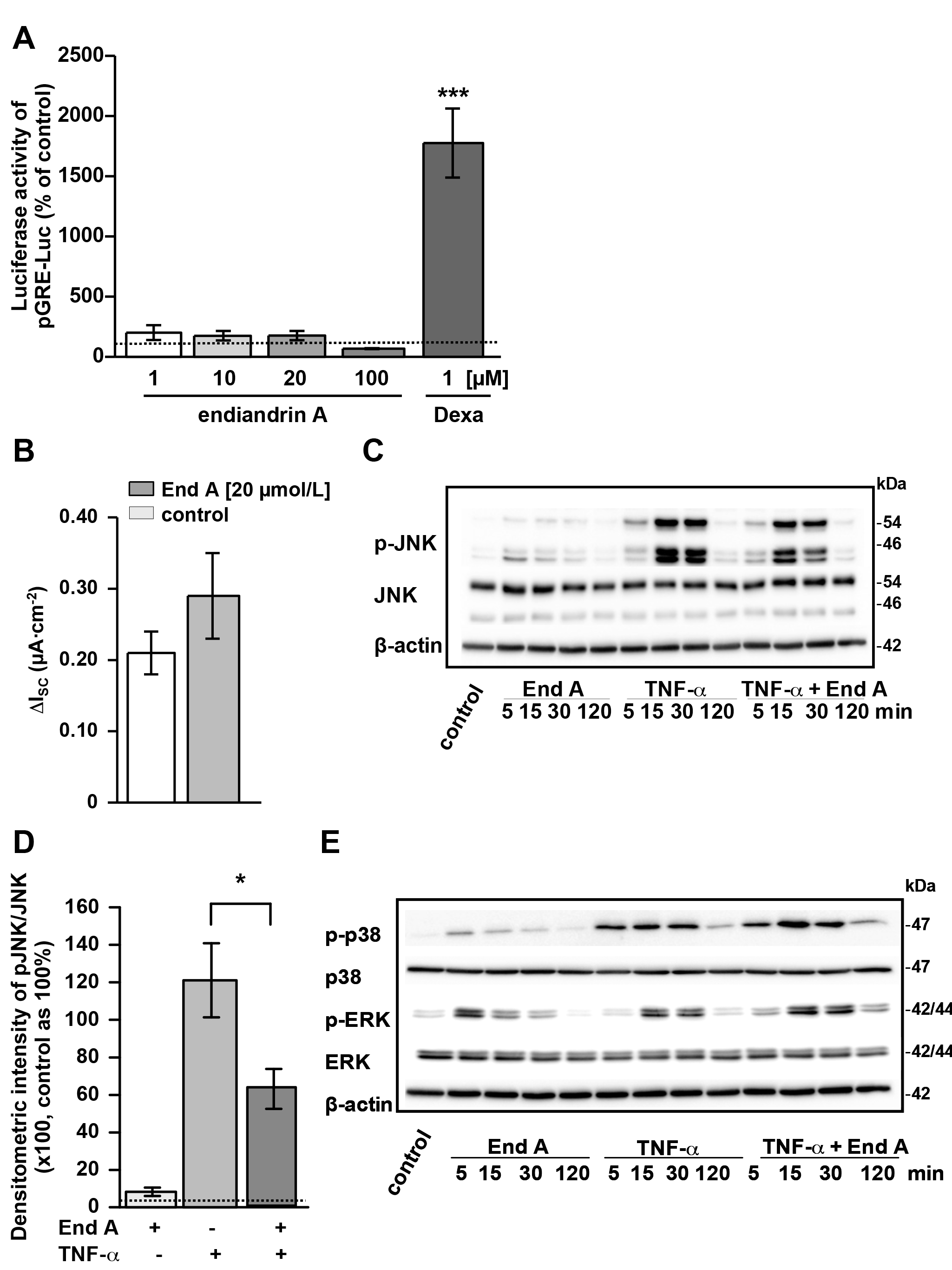

Supplement: Figure S1 — ( Fig. S1A ) HT-29/B6-GR cells were transfected with pGRE-Luc and incubated with endiandrin A (1, 10, 20 and 100 µM) for 24 hours. Incubation with dexamethasone (1 µM) alone was performed as positive control. Data are given as normalized relative luciferase activity and as means ± s.e.m., n = 4–6. ( Fig. S1B ) Measurement of ENaC-dependent Na+ absorption was determined as the drop in ISC after amiloride (100 µM). HT-29/B6-GR cells were incubated with endiandrin A (20 µM) for 48 hours. Data are means ± s.e.m., n = 6–12. ( Fig. S1C ) Western blot analysis of JNK and pJNK protein (∼46/54 kDa) of HT-29/B6-GR cell lysates incubated with TNF-α (500 IU/ml) and/or endiandrin A (20 µM) without dexamethasone for indicated times. Human β-actin (∼42 kDa) served as a loading control. ( Fig. S1D ) Densitometry of endiandrin A-induced effects on phosphorylated JNK levels normalized to total JNK. HT-29/B6-GR cells were incubated with TNF-α (500 IU/ml) and/or endiandrin A (20 µM) for 15 minutes. Shown are means ± s.e.m., n = 5, *P<0.05 compared to TNF-α exposure. ( Fig. S1E ) Western blot analysis of p38 and pp38 (∼38 kDa) as well as ERK and p-ERK MAPK protein (∼42/44 kDa) of HT-29/B6-GR cell lysates incubated with TNF-α (500 IU/ml) and/or endiandrin A (20 µM) without dexamethasone for indicated time points. Human β-actin (∼42 kDa) served as a loading control. (TIF) [file pone.0049426.s001.tif]
